# Supplementary material for: Computationally designed haemagglutinin with nanocage plug-and-display elicits pan-H5 influenza vaccine responses
Source: Emerg Microbes Infect. 2025 Jun 6;14(1):2511132. doi: 10.1080/22221751.2025.2511132 (PMC12258178; doi:10.1080/22221751.2025.2511132)
Supplement: Supplemental Material [file TEMI_A_2511132_SM7886.pdf]

Supplementary Information

Computationally designed haemagglutinin with nanocage plug-and-display elicits pan-H5 influenza neutralisation

Chloe Qingzhou Huang[1#], Rory A. Hills[2,3#], George W. Carnell[1,4], Sneha Vishwanath[1], Ernest T. Aguinam[1,5], Andrew C.Y. Chan[1], Phil Palmer[1], Laura O'Reilly[1], Paul Tonks[1], Nigel Temperton[6], Simon D.W. Frost[7,8,9,10], Laurence S. Tiley[7*], Mark R. Howarth[2*], Jonathan L. Heeney[1,10*]

[1]Laboratory of Viral Zoonotics, Department of Veterinary Medicine, University of Cambridge, Madingley Road, Cambridge, CB3 0ES, UK.
[2]Department of Pharmacology, University of Cambridge, Tennis Court Road, Cambridge, CB2 1PD, UK.
[3]Department of Biochemistry, University of Oxford, South Parks Road, Oxford, OX1 3QU, UK.
[4]One Virology, Wolfson Centre for Global Virus Research, School of Veterinary Medicine and Science, Sutton Bonington Campus, University of Nottingham, College Road, Loughborough LE12 5RD, UK.
[5]Wyss Institute for Biologically Inspired Engineering, Harvard University, 201 Brookline Ave Boston, MA 02115, USA.
[6]Viral Pseudotype Unit, Medway School of Pharmacy, The Universities of Kent and Greenwich at Medway, Central Avenue, Chatham, ME4 4TB, UK.
[7]Department of Veterinary Medicine, University of Cambridge, Madingley Road, Cambridge, CB3 0ES, UK.
[8]Department of Infectious Disease Epidemiology and Dynamics, London School of Hygiene and Tropical Medicine, Keppel Street, London, WC1E 7HT, UK.
[9]Microsoft Health Premonition, One Microsoft Way, Redmond, WA, 98052, USA.
[10]DIOSynVax Ltd, Janelle House, 6 Hartham Lane, Hertford, SG14 1QN, UK.

#These authors contributed equally.

*Corresponding authors
Jonathan L. Heeney: jlh66@cam.ac.uk
Mark R. Howarth: mh2186@cam.ac.uk
Laurence S. Tiley: lst21@cam.ac.uk

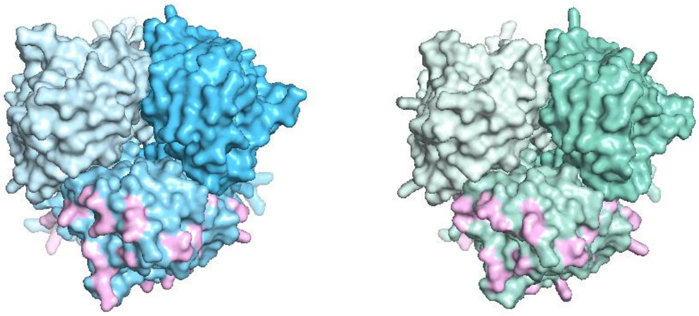

Supplementary Figure 1. Top-down views of HA antigens.
Conservation of residues between full-length DIOSvax-H5$_{inter}$ (H5DIOS) (blue) and H5Ast20 (green) mapped onto the van der Waals surface of HA homotrimers. Residue substitutions are represented in pink. Structural prediction was performed by AlphaFold 3 and displayed using PyMOL.

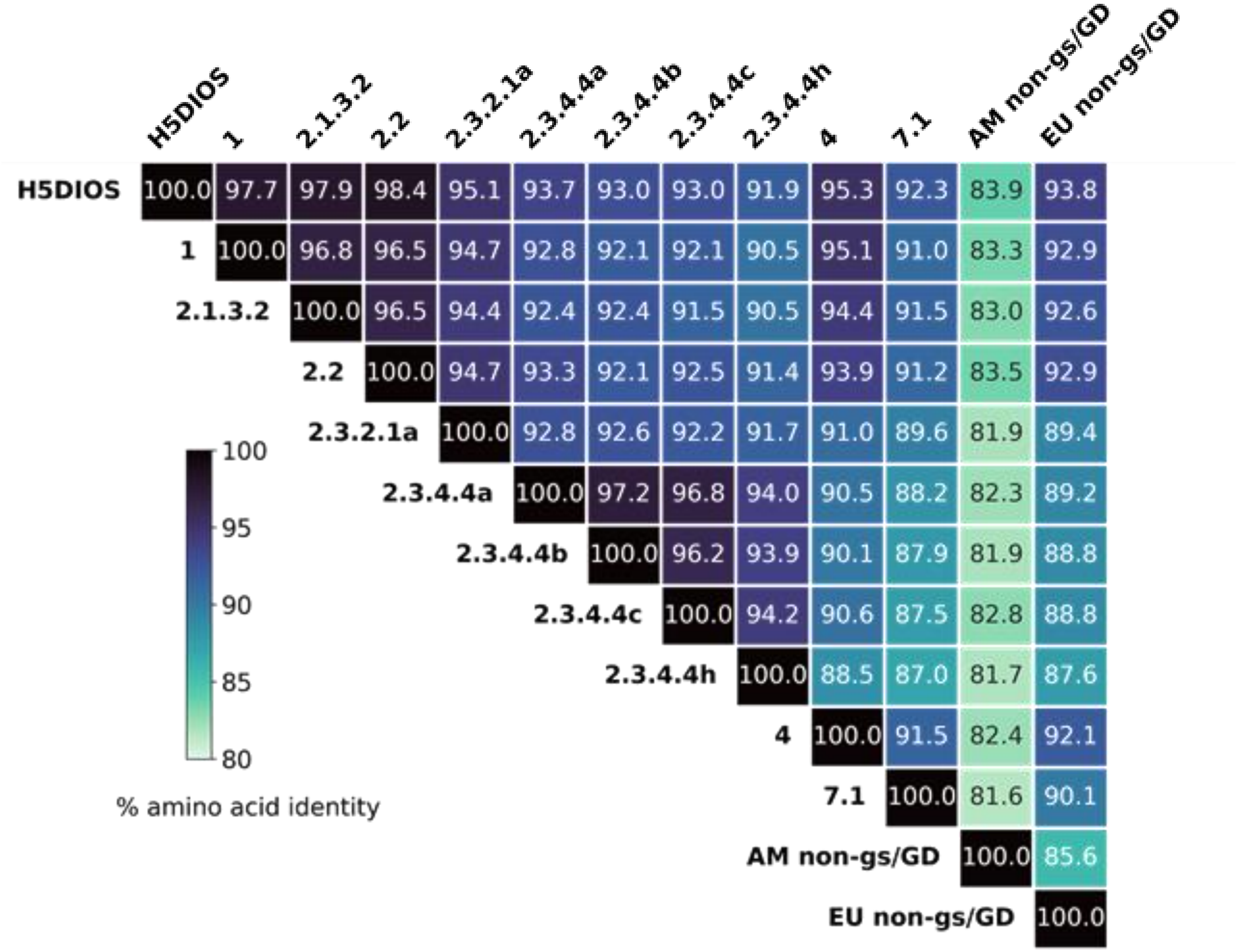

Supplementary Figure 2. H5 Residue Conservation.
Heat map of percent HA amino acid sequence identity of H5 viruses used in this study.

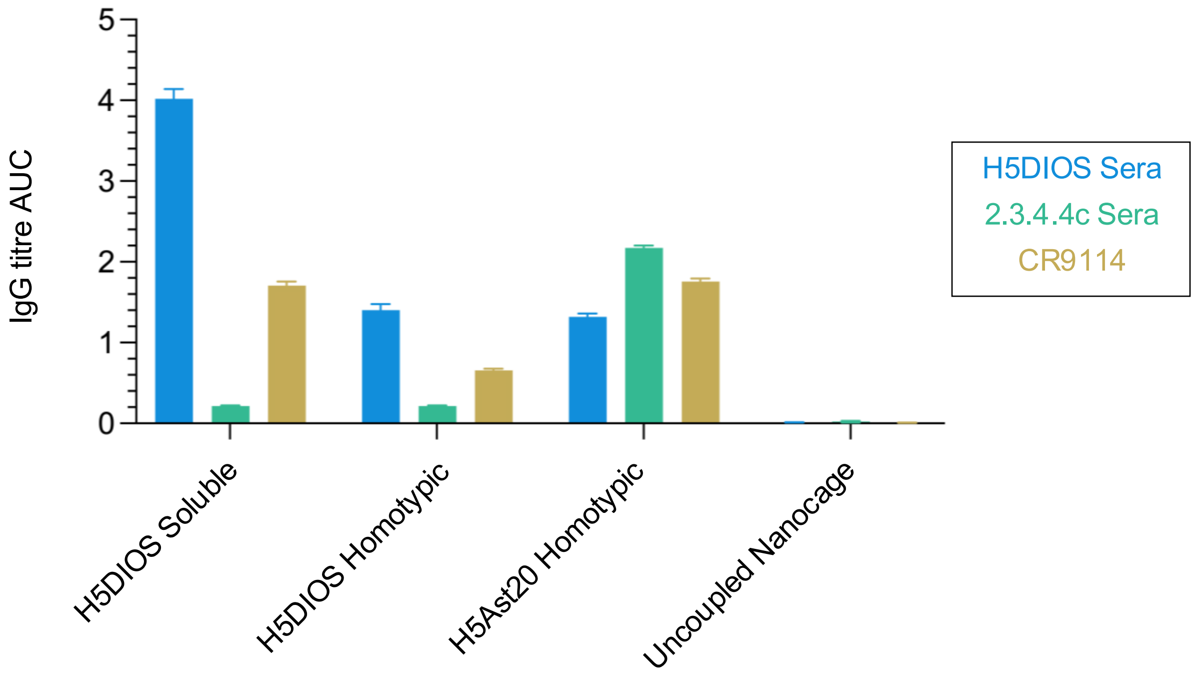

Supplementary Figure 3. Validation of antigenicity of vaccine antigens by ELISA. Binding of immobilised antigens to the broadly neutralising antibody CR9114 (yellow) and sera from mice previously immunised with DIOSvax-$H5_{inter}$ (H5DIOS) (blue) and 2.3.4.4c HA (green) as DNA vaccines. The antigens tested were DIOSvax-$H5_{inter}$ Soluble, H5DIOS Homotypic Nanocage, H5Ast20 Homotypic Nanocage and Uncoupled Nanocage. Results are represented as area under the curve (AUC) of a serial dilution. The mean is denoted by a bar ± 1 s.d.; $n = 2$.

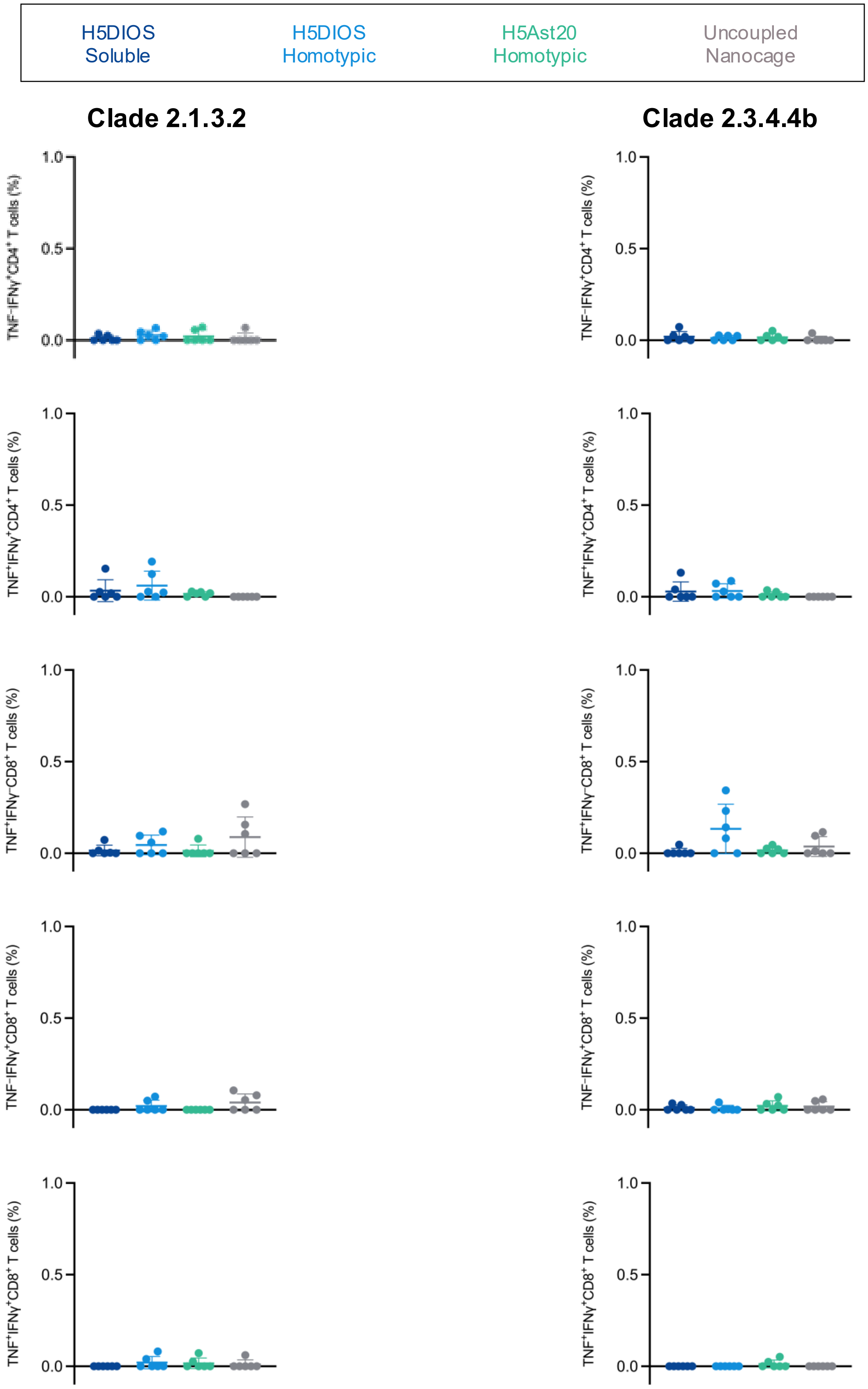

Supplementary Figure 4. Further Intracellular cytokine staining.

HPAI A/H5 HA-specific T cells from mice immunised with DIOSvax-H5$_{inter}$ Soluble (dark blue), DIOSvax-H5$_{inter}$ Homotypic Nanocage (light blue), H5Ast20 Homotypic Nanocage (green) or Uncoupled Nanocage (grey) underwent intracellular cytokine staining. Splenocytes were stimulated ex vivo with peptide pools for the HA of either the A/Indonesia/CDC835/2006 or A/Aves/Guanajuato/CPA-18539-23/2023 strain. Each dot represents one mouse. The mean is denoted by a bar ± 1 s.d.; $n = 6$. Statistical significance was calculated by ANOVA, followed by Tukey's multiple comparison post hoc test of % gated cells. All comparisons were non-significant.
